# Amorphous curcumin-based hydrogels to reduce the incidence of post-surgical intrauterine adhesions

**DOI:** 10.1093/rb/rbae043

**Published:** 2024-04-24

**Authors:** Wenya Zhang, Yuxin He, Yun Chu, Yuanxin Zhai, Song Qian, Xinhui Wang, Pengju Jiang, Pengfei Cui, Yin Zhang, Jianhao Wang

**Affiliations:** School of Pharmacy, Changzhou University, Changzhou 213164, P. R. China; School of Pharmacy, Changzhou University, Changzhou 213164, P. R. China; Jiangsu Trautec Medical Technology Co., Ltd, Changzhou 213200, P. R. China; Jiangsu Trautec Medical Technology Co., Ltd, Changzhou 213200, P. R. China; Jiangsu Trautec Medical Technology Co., Ltd, Changzhou 213200, P. R. China; School of Pharmacy, Changzhou University, Changzhou 213164, P. R. China; Jiangsu Trautec Medical Technology Co., Ltd, Changzhou 213200, P. R. China; School of Pharmacy, Changzhou University, Changzhou 213164, P. R. China; School of Pharmacy, Changzhou University, Changzhou 213164, P. R. China; Department of Gynecology, Changzhou Traditional Chinese Medicine Hospital, Changzhou 213004, P. R. China; School of Pharmacy, Changzhou University, Changzhou 213164, P. R. China

**Keywords:** intrauterine adhesion, curcumin, injectable hydrogel, regeneration of damaged endometrium, anti-fibrosis

## Abstract

The incidence of intrauterine adhesions (IUA) has increased with the rising utilization of intrauterine surgery. The postoperative physical barrier methods commonly used, such as balloons and other fillers, have limited effectiveness and may even cause further damage to the remaining endometrial tissue. Herein, we developed an injectable thermosensitive hydrogel using Pluronic F127/F68 as pharmaceutical excipients and curcumin as a natural active molecule. The hydrogel effectively addresses solubility and low bioavailability issues associated with curcumin. *In vitro*, drug release assays revealed that the amorphous curcumin hydrogel promotes dissolution and sustained release of curcumin. *In vitro* experiments reveal high biocompatibility of the hydrogel and its ability to enhance vascular formation while inhibiting the expression of fibrotic factor TGF-β1. To assess the effectiveness of preventing IUAs, *in vivo* experiments were conducted using IUA rats and compared with a class III medical device, a new-crosslinked hyaluronic acid (NCHA) gel. According to the study, curcumin hydrogel is more effective than the NCHA group in improving the regeneration of the endometrium, increasing the blood supply to the endometrium and reducing the abnormal deposition of fibrin, thus preventing IUA more effectively. This study provides a promising strategy for treating and preventing IUA.

## Graphical abstract

**Figure:**
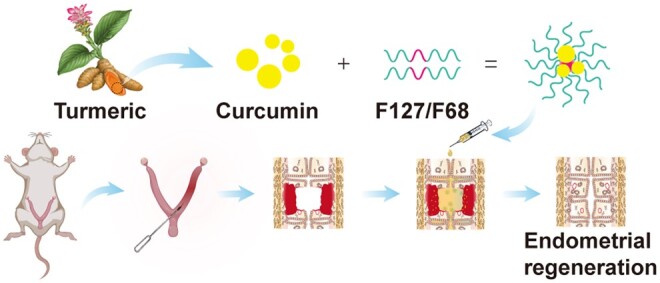

## Introduction

In normal physiological conditions, the uterine endometrium undergoes regular shedding and proliferation regulated by ovarian hormones, maintaining an appropriate shape and size of the uterine cavity [1]. Intrauterine adhesion (IUA) occurs if the endometrium fails to repair itself after damage, resulting in the growth of fibrous connective tissue and adhesion between anterior and posterior walls, which can lead to narrowing or complete closure of the cavity. IUA is a frequently occurring gynecological condition that can cause amenorrhea, cyclic abdominal pain, infertility, repeated miscarriages and preterm births, significantly impacting the physical and mental health of women [2–4].

**Figure 1.**
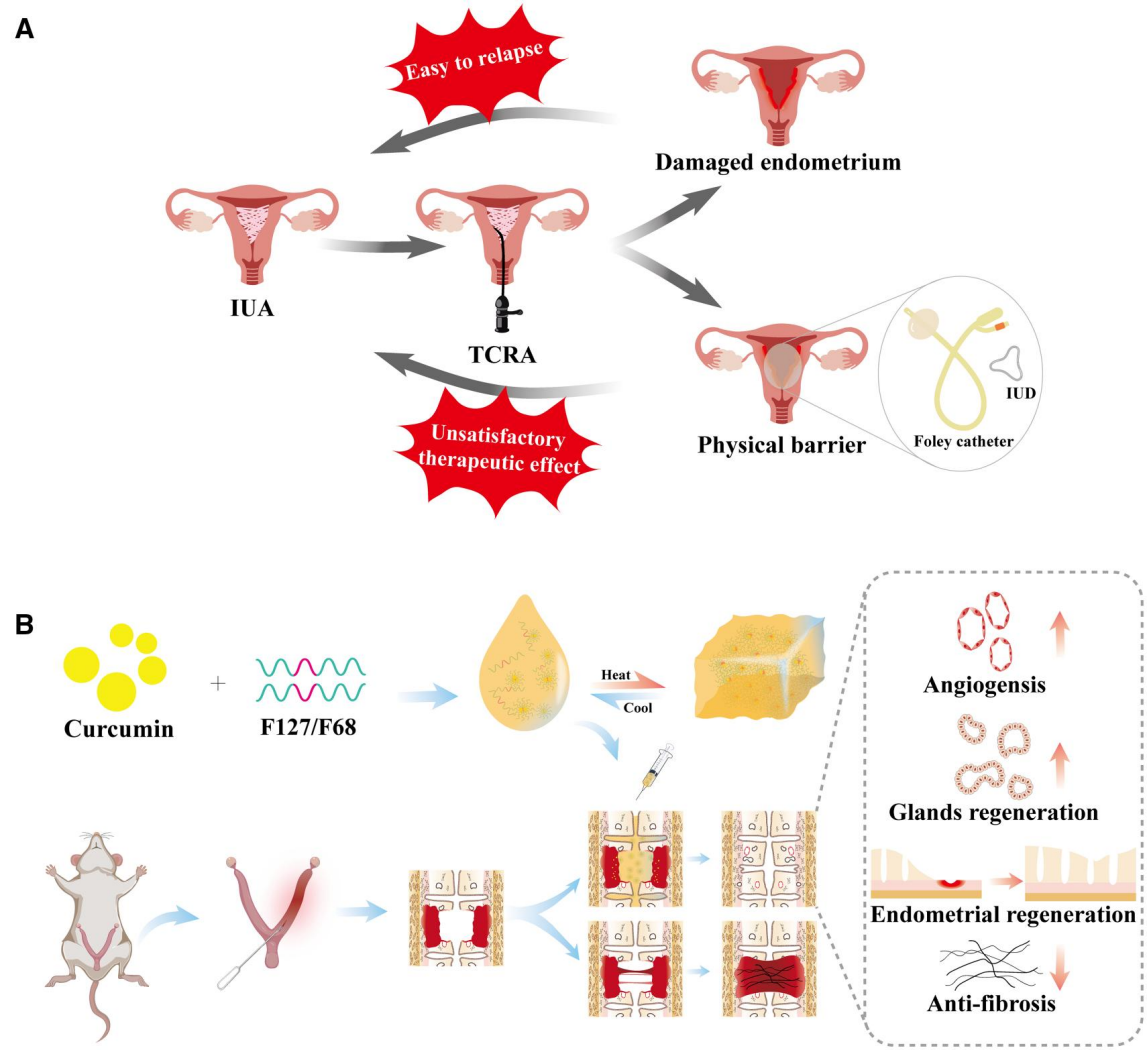
Schematic representation of the characteristics of Cur-gel and its application in endometrial repair and postoperative prevention of uterine adhesions. (**A**) TCRA is a common treatment for IUAs; however, there is a significant risk of adhesion recurrence following surgery. (**B**) IUA occurs when the impaired endometrium receives no treatment. However, by injecting Cur-gel into the uterus, curcumin is continuously released, which promotes new blood vessel formation, facilitates endometrial regeneration and reduces connective tissue production, ultimately preventing IUA.

Currently, the most effective treatment for IUA is transcervical resection of adhesions (TCRA) performed under hysteroscopy, which involves removing the adhesion tissue and restoring the uterine cavity’s shape [5]. However, postoperative recurrence is common, with a recurrence rate of up to 60% in patients with moderate to severe uterine adhesions [6]. Repeat surgeries are challenging to achieve satisfactory therapeutic effects and may even worsen the condition by causing further damage to the residual endometrial tissue [7]. The primary challenge in treating this disease is finding effective methods to prevent re-adhesion of the uterine cavity and avoid recurrent episodes. In clinical practice, physical barriers such as intrauterine devices, balloon catheters, or anti-adhesion agents are commonly used to prevent adhesion formation. These barriers isolate damaged tissues, prevent abnormal proliferative tissues from connecting, and to some extent, inhibit adhesion formation. However, when the barriers degrade or disappear, adjacent abnormal fibrous tissues may re-adhere, resulting in unsatisfactory treatment outcomes [8, 9] (Figure 1A).

Therefore, developing a safe and efficient anti-adhesion agent for the uterine cavity is urgently needed.

Curcumin (Cur), a polyphenolic compound extracted from turmeric and other plants, has gained considerable attention for its long-term safety and remarkable anti-inflammatory and antioxidant properties [10, 11]. The two main pathological manifestations of IUAs are abnormal fibrosis of the endometrium and decreased endometrial receptivity (caused by obstructed endometrial regeneration and reduced endometrial blood supply). Studies have shown that curcumin has substantial therapeutic effects in treating various fibrotic diseases, including liver and heart fibrosis [12–14], and promoting angiogenesis in wound healing [15–17]. Our interest lies in evaluating the potential of curcumin in alleviating endometrial fibrosis and its application for treating IUAs. However, the pharmaceutical applications of curcumin are significantly limited by its poor water solubility and low bioavailability. Numerous studies focus on enhancing the solubility and bioavailability of curcumin [18]. These methods encompass various approaches such as nanoparticles [19], nanoemulsions [20], cyclodextrins [21], phospholipid complexes [22], liposomes [23], nanogels [24] and self-assemblies [25]. Nevertheless, these methods possess certain drawbacks, including high cost, complex manufacturing processes, low drug loading, cytotoxicity and the need for organic solvents.

F127/F68 is a polymer composed of polyethylene oxide-polypropylene oxide-polyethylene oxide (PEO-PPO-PEO). Due to its non-toxicity and biocompatibility, it has received approval from the US Food and Drug Administration (FDA) for use in drug delivery applications [26]. As a non-ionic surfactant, F127/F68 significantly improves the solubility of curcumin, enabling its dispersion in an amorphous state within the polymer [27]. Additionally, when this copolymer is heated to a transition temperature, the PPO segment undergoes dehydration and crosslinks with the PEO segment to form spherical microgels, which subsequently interconnect to create a three-dimensional networked hydrogel [28].

A new type of sodium hyaluronate gel called New-crosslinked sodium hyaluronate gel (NCHA) has been developed as a class III medical device product for use after IUA surgery. It has been clinically proven to have anti-inflammatory effects and can effectively reduce the formation of postoperative IUAs [29, 30]. In a study to verify the therapeutic effects of Cur gel, both Cur gel and NCHA were implanted into rats with IUA and tissue evaluation was performed.

Consequently, we have developed a curcumin hydrogel called Cur-gel, which exists in a liquid state at room temperature for injection into the uterine cavity, and undergoes gelation at physiological temperature, continuously releasing curcumin. It enhances endometrial recovery and markedly reduces fibronectin deposition (Figure 1B). The hydrogel preparation is simple, adjustable and more effective than existing anti-adhesion agents. It has great potential for use in preventing IUAs after surgery.

## Materials and methods

### Materials

Curcumin (purity higher than 98%) and DMEM medium were purchased from Shanghai Adamas Reagent Co., Ltd Pluronic^®^F127 (F127) and Pluronic^®^F68 (F68) were purchased from BASF SE (Germany). Growth factor-reduced Matrigel was purchased from Corning Incorporated (USA). Recombinant Bovine bFGF, Trizol and BCA Protein Assay Kit were purchased from Beyotime Biotechnology. hESC and HUVEC cell lines were purchased from ATCC. Thiazolyl Blue (MTT, purity higher than 98%) was purchased from Biofroxx (Germany), DMSO was purchased from Aladdin Reagent Shanghai Co., Ltd and Endothelial Cell Medium (ECM) was purchased from ScienCell (USA). Fetal bovine serum (FBS) was purchased from Zhejiang Tianhang Biotechnology Co., Ltd anti-TGF-β1 (21898-1-AP, Proteintech, Rosemont, IL, USA). Cyanine7 dye was synthesized in our laboratory. Cross-linked sodium hyaluronate gel for the uterine cavity (HA-gel) was purchased from Changzhou Bioregen Biomedicine Co., Ltd.

### Preparation of F127/F68 thermosensitive hydrogels

Different amounts of F127 and F68 were dissolved in sterile physiological saline and gently mixed using magnetic stirrers for 24 h at 4°C until complete dissolution of all Pluronic granules and the attainment of a clear solution. The thermosensitive sol-gel transition was assessed using the small tube inversion method. The samples were transferred to small penicillin bottles, and each polymer’s temperature range was analyzed, spanning from 15°C to 37°C. The vials were initially equilibrated in a 15°C water bath for 10 min and then heated at a rate of 1°C per 5 min. The gel temperature was determined 1 min after the tube had been inverted. To construct *in situ* gelling matrices, the gelation temperature should fall within the range of 26°C to 36°C, with polymer concentrations not exceeding 30 wt%. Consequently, a F127/F68 weight ratio of 20/6 was chosen for subsequent experiments.

### Preparation of Cur@F127/F68 thermosensitive hydrogels

Different amounts of curcumin powder were weighed and poured into a certain amount of F127/F68 hydrogel, which was fully stirred at room temperature for 24 h to see the dissolution of curcumin, and 0.15 and 1 mg/ml Cur@P407/P188 hydrogel were determined for the follow-up experiments.

### Micromorphology of thermosensitive hydrogels

The morphology, pore size and interconnectivity of blank hydrogels and drug-loaded hydrogels were assessed using a Scanning Electron Microscope (SEM; Hitachi, Regulus-8100, Japan). The hydrogel was frozen at −80°C and then lyophilized. Freeze-dried cross-sections of the powder were sprayed with gold and subsequently analyzed through scanning.

### XRD spectra analyses

Amorphous and crystalline phases were specified using X-ray diffraction (XRD) analysis. The measurement was recorded on an X-ray diffractometer (Bruker, D8 Advance, Italy) and the samples were analyzed in the range between 5°and 60° 2θ with step angle of 0.02°.

### Differential scanning calorimetry

Accurate measurements of curcumin (Cur), freeze-dried blank hydrogels and freeze-dried drug-loaded hydrogels, each weighing 5 ± 0.5 mg, were taken and placed in sealed aluminum trays. Prior to differential scanning calorimetry (DSC) analysis, the aluminum trays were covered and pierced. DSC analysis was conducted using a differential scanning calorimeter (Hitach, DSC 7020, Japan) with nitrogen gas used as the flushing agent for all experiments. The temperature was conventionally raised from 20°C to 250°C for the DSC procedure.

### Rheological evaluation

Rheological measurements were conducted using a rotary rheometer (Malvern, Kinexus Pro, Britain) fitted with concentric cylinders and connected to a circulating water bath. The rheological characteristics of the hydrogel at physiological temperature were examined through amplitude scanning at 37°C. To assess the rheological properties of temperature-sensitive hydrogels, a constant shear strain (0.5%) and a constant frequency (1 Hz) were employed. The storage modulus *G*′ and loss modulus *G*′′ of the hydrogels were measured while the temperature increased from 0°C to 50°C at a rate of 5°C/min.

### Release study of curcumin *in vitro*

To begin the experiment, take 1 ml of drug-loaded hydrogel and put it in a 15 ml centrifuge tube. Place the tube in a constant temperature shaker at 37°C (120 rpm) and wait until the hydrogel has turned into a gel. Once it has turned into a gel, add 5 ml of isothermal phosphate buffer (PBS). At a specific time point, remove 4 ml of the release solution and replace it with an equal amount of PBS. Use an ultraviolet spectrophotometer to determine the concentration of curcumin, and then calculate the cumulative release rate of curcumin.

### Hemolysis assay

To evaluate the compatibility of hydrogels with the blood, the rate of hemolysis was measured. The hemolysis rate was determined as follows: Rat blood (500 μl) was centrifuged at 2000 rpm for 15 min to separate red blood cells (RBCs) from serum. The resulting RBC sediment was washed thrice with PBS buffer until a clear supernatant was observed. The RBC sediment was then diluted with PBS to a concentration of 5% (v/v) for later use. For the positive and negative controls, RBCs were incubated with deionized water and PBS, respectively. To each 500 μl of the 5% RBC suspension, 500 μl of blank hydrogel and drug-loaded hydrogel (1 mg/ml Cur-gel) were added. The RBC suspension samples prepared as mentioned above were incubated at 37°C for 1 h, followed by centrifugation at 2000 rpm for 15 min. A digital camera captured the image of each centrifuged sample, and the absorbance of each supernatant was measured at 540 nm using a plate reader. Finally, the hemolysis rates were calculated using the following formula:

$$\text{hemolysis}\;\text{rate}\;(\%)=\frac{{\text{OD}}_{T}-{\text{OD}}_{N}}{{\text{OD}}_{P}-{\text{OD}}_{N}}\times 100$$

where OD_T_, OD_N_ and OD_P_ are absorbance values of the test group (hydrogels group), negative group and positive group, respectively. Additionally, RBCs treated with the experimental sample set were observed for any morphological changes. After incubation at 37°C for 1 h, the RBC solutions were centrifuged at 20 000 rpm for 15 min. The collected RBC pellets were then diluted in PBS, dispensed onto clean glass slides, covered with a coverslip and finally photographed using a microscope equipped with a digital camera.

### Cytotoxicity test

MTT (Thiazolyl Blue) assay was utilized to evaluate the cytocompatibility of hydrogels. The hydrogel sample was extracted with DMEM medium at a ratio of 0.2 g/ml and incubated in a 37°C incubator for 24, 48 and 72 h, respectively. Exponentially growing human endometrial stromal cells (hESC) and human umbilical vein epithelial cells (HUVEC) were seeded in a 96-well plate at a concentration of 5000 cells/well. After 24 h of incubation, the culture media for hESC and HUVEC were replaced with the extracted hydrogel fluid. The culture medium was employed as the negative control. Then the plates were incubated at 37 ± 1°C for 24 h in a 5% CO_2_ atmosphere. Subsequently, 20 μl of MTT was added to each well and incubated for 4 h. Next, the MTT dye was removed, and 150 µl of DMSO was added. The analysis was conducted using a plate reader at a wavelength setting of 490 nm. The cell viability was calculated using the following formula:

$$\text{Cell}\;\text{viability}\;(\%)=\frac{\text{OD}490(\text{sample})\;}{\text{OD}490\;(\text{control})\;}\times 100$$

### Tube formation assay

Before use, thaw an appropriate volume of Matrigel with reduced growth factors at 4°C for 24 h. Add 50 µl of Matrigel with reduced growth factors to each well of a pre-chilled 96-well plate on ice. Allow polymerization to occur for 1 h on a horizontal surface in a 37°C incubator.

Digest the grown HUVEC with trypsin, suspend it in serum-containing ECM medium, centrifuge at room temperature at 1200 rpm for 3 min to pellet the cells, then resuspend it with 2 ml of serum-containing ECM medium, and determine HUVEC concentration using a cell counter. Transfer an appropriate volume of HUVEC cell suspension into a 1.5-ml centrifuge tube, centrifuge it at 3000 rpm for 3 min at room temperature, remove as much supernatant as possible, and subsequently add curcumin samples with varying concentrations to the centrifuge tube for resuspension. Retrieve the Matrigel-coated 96-well plate, inoculate 1 × 10^4^ cells per well, and include three parallel wells for each sample. Negative control consists of ECM complete medium, while positive control contains ECM complete medium with a 3-ng/ml bFGF concentration. Incubate the plate in an incubator, remove it for observation and capture images at a specific time.

### Western bolt

hESCs were incubated with curcumin samples. After 24 h, the samples were removed, and the cells were washed three times with cold PBS. The cells were separated using a scraper, collected in a 1.5-ml centrifuge tube and centrifuged at 3000 rpm at 4°C for 5 min. After removing the supernatant, 250 μl of RIPA lysate (containing protease and phosphatase inhibitors) was added and the cells were lysed on ice for 30 min. The cell lysate was centrifuged at 12 000 rpm for 15 min at 4°C. Then, the supernatant was collected, and the protein concentration was determined using a BCA detection kit. Equal amounts of protein were subjected to electrophoresis on a 15% SDS-PAGE gel and then transferred to a PVDF membrane. After blocking the membrane with 5% skimmed milk powder for 1 h at room temperature, it was incubated with the TGF-β1 antibody overnight at 4°C. The membrane was washed three times with TBST and then incubated with horseradish peroxidase-labeled goat anti-rabbit IgG for 1 h at room temperature. Anti-β-actin antibody was used as a protein loading control. Quantitative analysis was performed using a Tanon 5200 Multi (Tanon, China) and was shown as the relative expression to β-actin.

### Construction of IUA models in rats

In this study, sexually mature female Sprague Dawley rats, aged 8 weeks and weighing 220–250 g, were obtained from the Shanghai Experimental Animal Center. The rats were cultured adaptively for one week. The estrous period was determined using the vaginal exfoliated cell smear method. Rats showing normal estrus were selected for model creation, and the IUA rat model was established using the curettage injury technique. Briefly, rats in estrus were anesthetized, and their abdominal cavities were opened to expose the Y-shaped uterus. A transverse incision of ∼2 mm was made 0.5 cm from the cervix of the two uterine horns. The endometrium was scraped using a self-made curettage spoon until the uterine cavity walls became rough and bled, and the uterine wall became thin and translucent. The uterine opening was then closed. Subsequently, the abdominal cavity was rinsed with normal saline, and the abdomen was sutured in layers. All animal experiments adhered to the guidelines for the care and use of laboratory animals at Changzhou University and received approval from the Animal Ethics Committee of Changzhou University (No. 2023030125).

### Delayed evaluation of drugs by hydrogels *in vivo*

To assess the delayed release of drugs enclosed in F127/F68 thermosensitive hydrogels *in vivo*, 100 μl of Cy7 saline solution and Cy7 hydrogel (Cy7 concentration of 1 mg/ml) were injected into the right uterine cavity of healthy rats. On Days 1, 2, 3 and 5 after administration, the rats were sacrificed, the uterus was collected and then the surface of the uterus was washed with saline. Finally, a small animal *in vivo* optical imaging system (Tanon ABL X 5) was used to collect fluorescence images of the intact uterus.

### Safety evaluation of hydrogel *in vivo*

The rats were carefully selected and assigned to three different groups using randomization. These groups included: (i) the sham surgery group, in which only a uterine incision was performed without curettage; (ii) the control group, consisting of rats with IUAs but no treatment; (iii) the HCur-gel group, 100 μl HCur-gel was injected into the uterus of rats. On the eighth day, the blood of rats was collected and the blood safety was evaluated.

### Implantation of hydrogels *in vivo*

The rats were carefully selected and assigned to six different groups using randomization. These groups included: (i) the sham surgery group, in which only a uterine incision was performed without curettage; (ii) the control group, consisting of rats with IUAs but no treatment; (iii) the Gel group, in which the IUA model was established and both uteruses were injected with 100 μl of blank hydrogel; (iv) the LCur-gel group, in which the IUA model was established and both uteruses were injected with 100 μl of 0.15 mg/ml Cur-gel; (v) the HCur-gel group, in which the IUA model was established and both uteruses were injected with 100 μl of 1 mg/ml Cur-gel; and (vi) the HA group, in which the IUA model was established and both uteruses were injected with 100 μl of cross-linked sodium hyaluronate gel (HA). Throughout the experiment, the estrus cycle of the rats was carefully monitored. At the end of two estrus cycles, the rats were sacrificed and their uteruses were removed.

### Evaluation of the number of endometrial glands and endometrial thickness

The uterine horns were fixed with a 4% paraformaldehyde tissue fixative. Next, the uterine horns were embedded in paraffin, sliced, dewaxed and hydrated at room temperature. Subsequently, HE staining was performed. The gland count and endometrial thickness were determined by analyzing the pathological images using image viewing software (NPD.view2). The endometrial thickness was measured in both the horizontal and vertical directions, and the average of these measurements was calculated.

### Immunohistochemical staining

The uterine horns were fixed in 4% paraformaldehyde tissue fixative, then embedded in paraffin, sliced, dewaxed and hydrated at room temperature. Immunohistochemical staining was performed using CD31 and TGF-β1 antibodies. The pathological images were processed using image browsing software (NPD.view2). Four visual fields were randomly selected at 40× and 80× magnification for analysis of the positive ratio using ImageJ software.

### Evaluation of anti-fibrosis effect

The uterine horns were fixed with a 4% paraformaldehyde tissue fixative. Subsequently, they were embedded in paraffin, sliced, dewaxed and hydrated at room temperature. The samples were subjected to Masson and Sirius red staining. Masson staining was analyzed using image browsing software (NPD.view2) on pathological images. Four visual fields were randomly selected at 80× magnification. Finally, the fibrosis ratio was evaluated using ImageJ software. The sections were stained with Sirius red and then photographed under polarized light.

### Statistical analysis

All experimental data are presented as mean ± standard deviation. Statistical significance for multiple comparisons was determined using one-way analysis of variance. Quantification of all dyed pictures was done using ImageJ, and statistical analysis was performed using GraphPad Prism 8 or Origin 2022. *P* < 0.05 was considered statistically significant.

## Results and discussion

### Preparation and characterization of thermosensitive hydrogels

**Figure 2.**
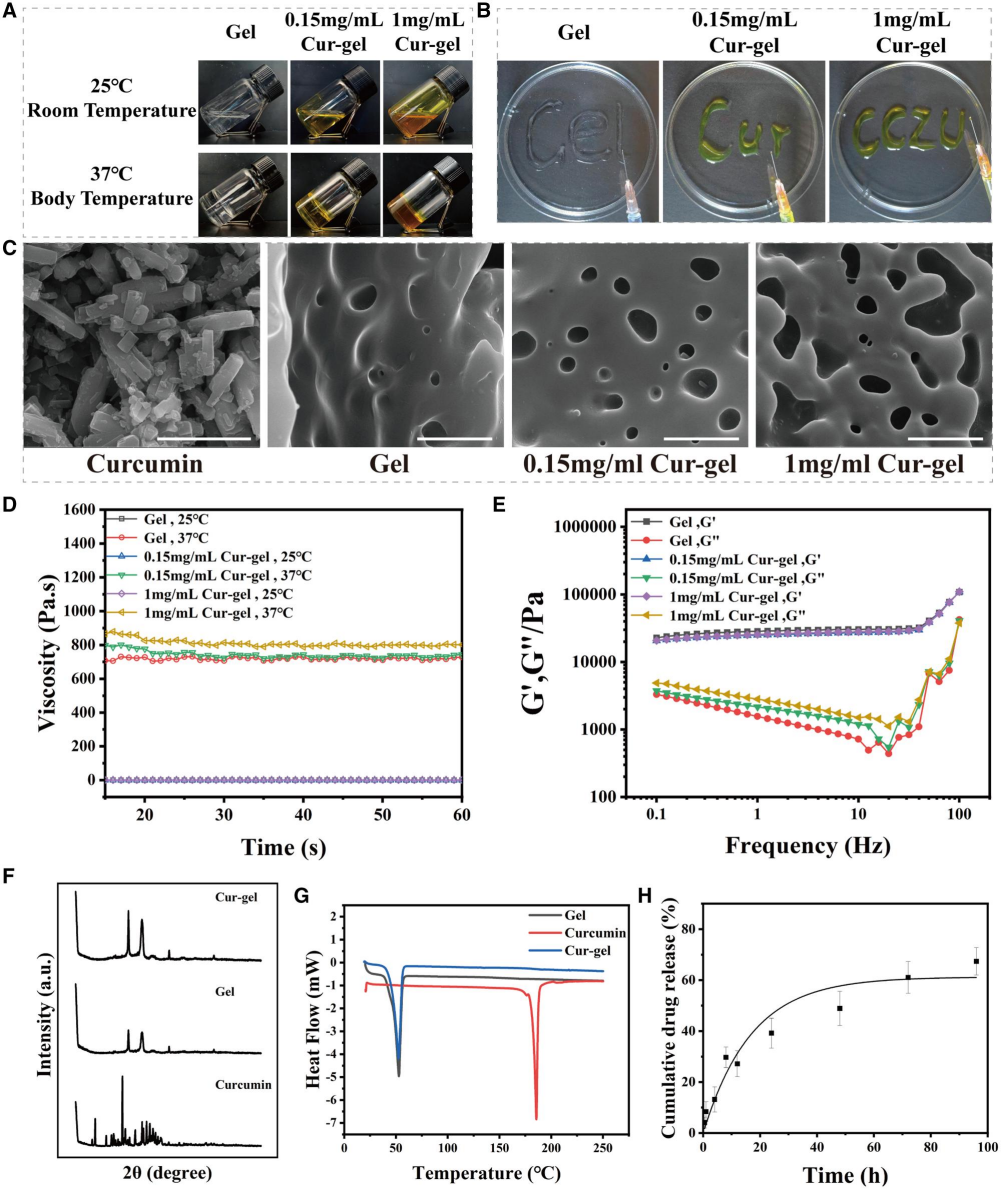
Fabrication and characterization of the gel and Cur-gel. (**A** and **B**) Photograph of the obtained gel and Cur-gels with thermosensitive and injectable properties. (**C**) Scanning electron microscopy (SEM) images of curcumin and hydrogels. Scale bar: 5 and 2 μm. (**D**) Viscosity of hydrogel at 25°C and 37°C. (**E**) Amplitude modulus of hydrogels at 37°C. The storage modulus (*G*′) is much larger than the loss modulus (*G*′′), indicating gelation of the hydrogel at this temperature. (**F** and **G**) XRD patterns and DSC thermograms of curcumin powder, gel freeze-dried powder, and Cur-gels freeze-dried powder demonstrated the amorphous nature of curcumin in the Cur-gels. (**H**) Cumulative curcumin release from cur-gels.

Thermosensitive hydrogels were prepared using different ratios of F127 and F68. To determine the optimal gelation temperature, various ratios were tested as presented in Table S1. Based on the experimental results, the final hydrogel system was determined to consist of F127 (20% wt) and F68 (6% wt). To measure the solubility of curcumin, it was dissolved in this hydrogel system (Figure S1). Determined to use two hydrogel formulations containing curcumin, with concentrations of 0.15 and 1 mg/ml, respectively, named LCur gel and HCur gel, for subsequent experiments. The hydrogel exhibited favorable thermosensitive properties and injectability, as depicted in Figure 2A and B.

Rheological analysis of the hydrogel revealed that the storage modulus (*G*′) rapidly surpassed the loss modulus (*G*′′) at the sol-gel transition temperature (Tsol-gel), indicating a phase transition from sol to gel (Figure S2). Under a frequency range of 1–100 at 37°C, *G*′ was consistently greater than *G*′′, indicating the formation of a relatively stable three-dimensional network structure with elastic behavior and solid-like rheological properties. As the frequency increased, *G*′′ gradually decreased, implying the shear-thinning behavior of the hydrogel, which is consistent with its injectability (Figure 2E). The viscosity of the hydrogel increases at body temperature, which will help hydrogel to stay in the uterus (Figure 2D). Finally, after freeze-drying, SEM analysis revealed that the hydrogel possessed a porous structure, while curcumin displayed a rod-shaped crystal structure (Figure 2C).

Poloxamer is a commonly used carrier for solid dispersants to improve the solubility of poorly soluble drugs [31]. Curcumin in F127/F68 can be encapsulated in the micellar nucleus, and the freeze-dried Cur-F127/F68 powder becomes an amorphous curcumin solid dispersant. XRD and DSC techniques were employed to evaluate the physical transformation of curcumin [22, 32]. The XRD analysis indicates the disappearance of the diffraction peaks associated with crystalline curcumin in the Cur-F127/F68 powder (Figure 2F). Furthermore, the DSC temperature graph demonstrates a prominent endothermic peak at ∼185°C, which corresponds to the melting point of the curcumin powder. In contrast, the F127/F68 powder exhibits an endothermic peak at around 52°C, signifying the melting temperature (*T*_m_) of F127/F68. Notably, no endothermic peak is observed in the vicinity of the melting point of individual crystalline curcumin in the Cur-F127/F68 powder (Figure 2G). These observations strongly suggest the amorphous nature of curcumin within the F127/F68 matrix, thereby facilitating its release. In contrast, pure curcumin, exhibits limited solubility in PBS, whereas Cur-gel shows rapid dissolution in PBS, thereby enabling a sustained release of curcumin and yielding superior therapeutic effects *in situ* (Figure 2H).

### Hemolysis assay and safety evaluation

**Figure 3.**
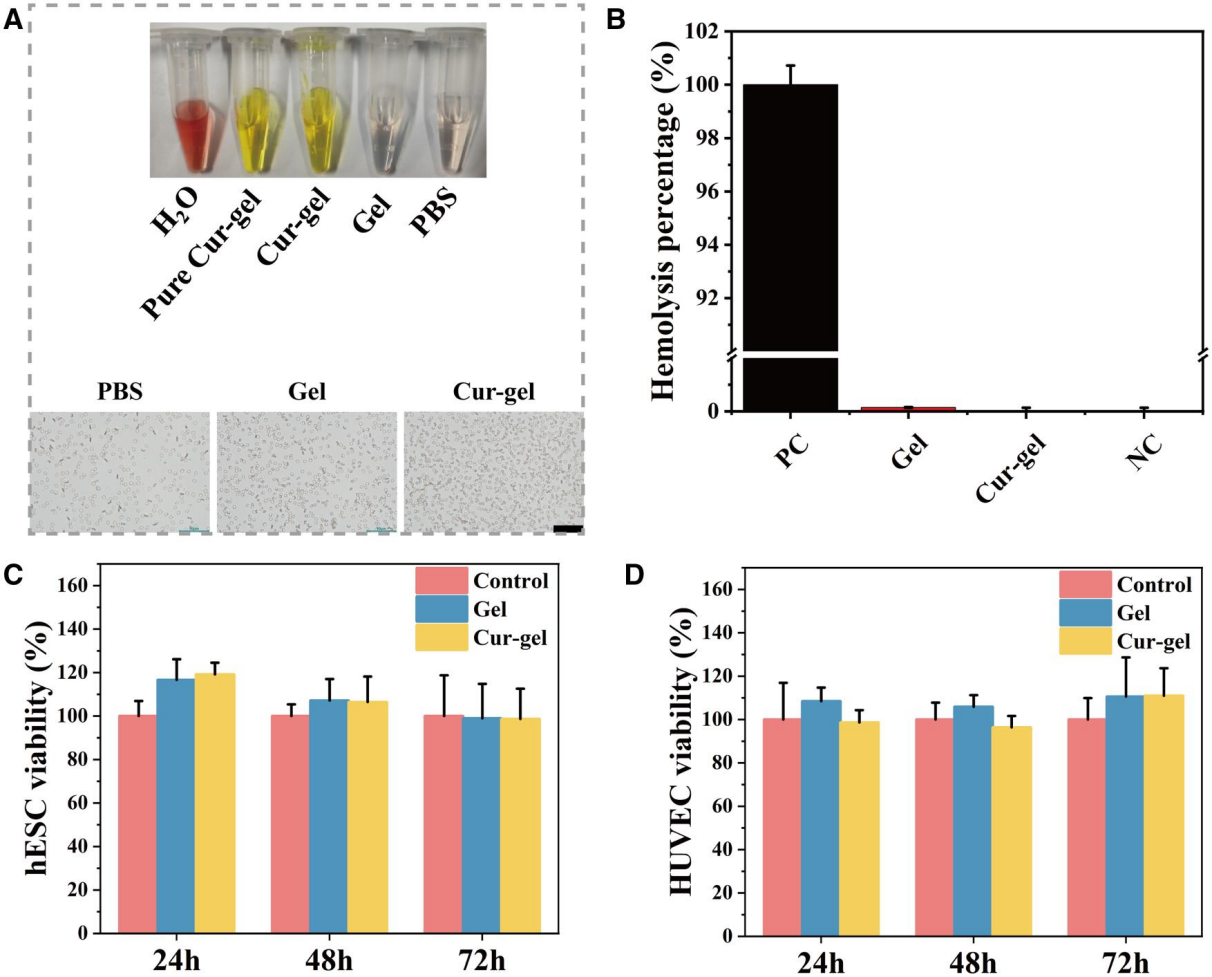
Biocompatibility assessment of hydrogels. (**A** and **B**) The hemolysis assay of hydrogels is used to evaluate their hemocompatibility. 0.1% Triton was used as a positive control, and PBS was used as a negative control. (**C** and **D**) The viability of hydrogels was assessed using MTT assay with hESC and HUVEC cells, respectively. Control cells were not exposed to any material extraction medium. Each value represents the mean ± SD (*n* = 3 per group).

Biocompatibility plays a crucial role in the development of medical anti-adhesive agents [33]. To evaluate the safety of the hydrogel, we conducted hemolysis tests, cell viability tests and blood safety test *in vivo*. Since the hydrogel will come into contact with damaged endometrial tissue and be exposed to blood, we performed a blood compatibility evaluation. The results, as shown in Figure 3A, indicate that the RBC status after treatment with Gel and Cur-gel is nearly identical to that after PBS treatment.

The hemolysis rate of Gel and Cur-gel is extremely low under the test conditions (Figure 3B), indicating excellent blood compatibility. As shown in Figure S9, the blood routine test of rats implanted with Cur-gel showed no significant changes compared to the sham operation rats and IUA rats. The main liver function markers, aspartate aminotransferase (AST) and alanine aminotransferase (ALT), and the renal function marker, uric acid (UA), did not show any significant changes. Thus, it can be concluded that curcumin hydrogel does not have any noticeable acute toxicity. Furthermore, when the hydrogel extracts were co-cultured with hESC and human umbilical vein endothelial cells (HUVEC), cell viability showed no significant difference compared to the control group (Figure 3C and D). These *in vitro* experiments suggest that the hydrogel might be a safe anti-adhesive agent for the uterine cavity.

### *In vitro* biological function of Cur@F127/F68 hydrogels

**Figure 4.**
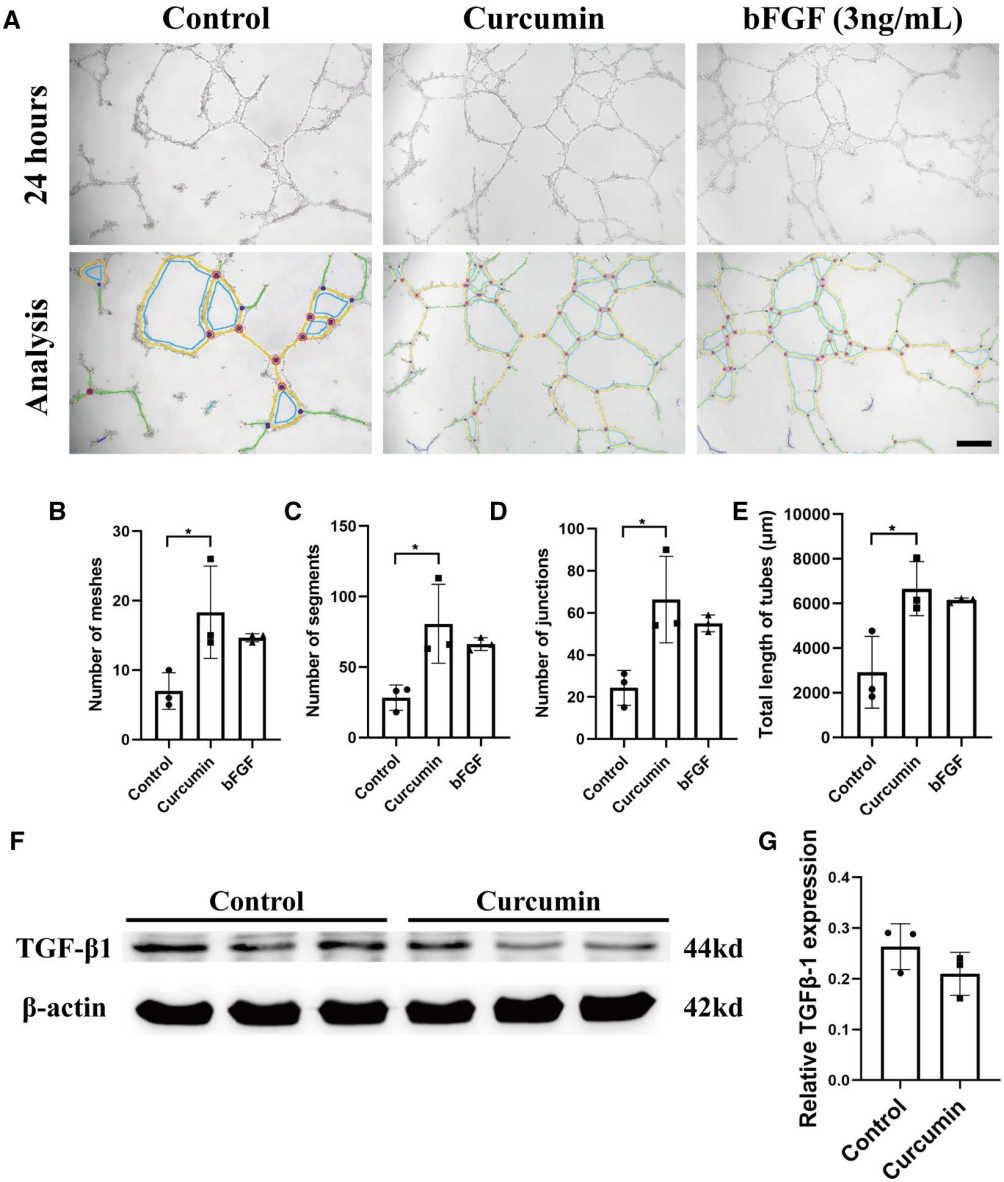
*In vitro* biological activity evaluation. (**A**) Measurement of angiogenesis with the tube formation assay. The photomicrographs were analyzed using Angiogenesis Analyzer (ImageJ). Scale bar: 100 μm. (**B**–**E**) Quantitative analysis of capillary tube formation parameters using the Angiogenesis Analyzer for ImageJ. The parameters include the number of meshes, the number of segments, the number of junctions and the total length of the tube. (**F**) Western blot analysis of hESC treated with curcumin for 24 h (blots were cropped and presented). (**G**) The quantification of (F) by ImageJ.

According to research, curcumin has been reported to regulate blood vessel formation and has the potential to promote blood vessel formation in wounds [10, 34]. To investigate this capability, an *in vitro* angiogenesis experiment was conducted. Initially, the effect of various concentrations of curcumin on the viability of HUVECs was explored, and the appropriate concentration of curcumin was determined (Figure S3). Subsequently, HUVECs were co-cultured with the determined concentration of curcumin for 8 h. Our observations indicate that the cell line exhibited a significantly greater tendency in network mesh count, segment count, junction count and total tube length compared to the control group (Figure S4). This tendency reached its highest significance level after co-culturing HUVECs for 24 h (Figure 4A–E).

Additionally, the expression of transforming growth factor β1 (TGF-β1), a crucial fibrotic factor associated with various fibrotic diseases, was assessed [35–37]. The expression of TGF-β1 in hESCs treated with curcumin was found to be lower than that in the control group (Figure 4F and G), implying that curcumin may possess effective anti-endometrial fibrosis properties.

### The therapeutic effects of Cur@F127/F68 hydrogels for IUA rats *in vivo*

**Figure 5.**
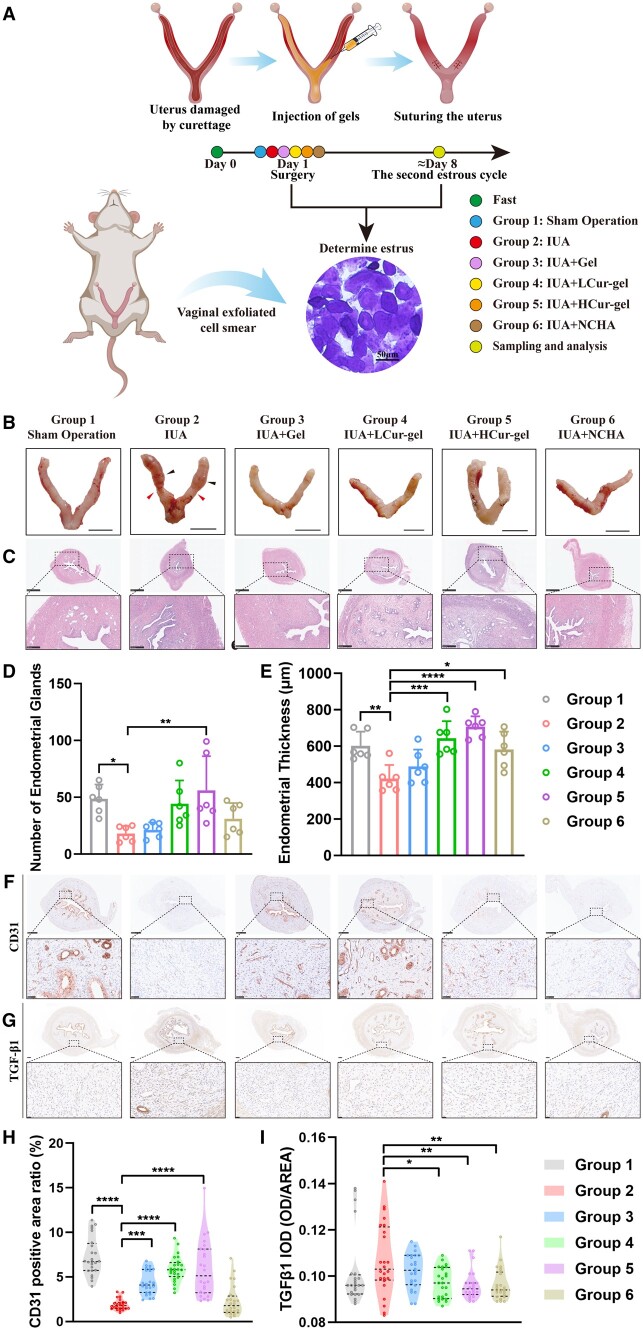
The therapeutic effects of Cur-gels in rats with IUA. (**A**) Schematic diagram and timeline outlining surgical procedures, with the grouping of distinct categories. (**B**) Photographic images of the uterus after treatment. Scale bar: 1 cm. (**C**) Corresponding HE staining. Scale bar: 1 mm, 250 μm. (**D**) Number of endometrial glands. (**E**) Endometrial thickness quantitative analysis. (**F**) Corresponding CD31 immunohistochemical staining. Scale bar: 500 μm, 50 μm. (**G**) Corresponding TGF-β1 immunohistochemical staining. Scale bar: 200 μm, 20 μm. (**H**) Quantitative analysis for CD31 protein expression levels. (**I**) Quantitative analysis for TGF-β1 protein expression levels.

Hydrogels can delay the retention of the encapsulated drug *in vivo* (Figure S5). The utilization of the IUA rat model aimed to assess the therapeutic efficacy of hydrogels *in vivo*. To minimize hormonal influences on the endometrium, the estrous cycle of rats was determined via vaginal smears [38, 39]. Subsequently, surgical procedures were conducted during the estrous phase. Euthanasia and sampling took place after two estrous cycles (Figure 5A).

The IUA group demonstrated substantial alterations in uterine tissue morphology, characterized by evident IUAs and the accumulation of fluid in the uterine cavity, as opposed to the sham surgery group. Nonetheless, the injection of Gel, Cur-gel and new-crosslinked hyaluronic acid gel (NCHA) exhibited specific anti-adhesion effects without inducing significant tissue morphology changes (Figure 5B). Hematoxylin-eosin (HE) staining of the sections revealed that the IUA group displayed considerable narrowing of the uterine cavity and adhesions. Conversely, the LCur-gel group and HCur-gel group demonstrated satisfactory uterine cavity morphology, an increased gland count and notably thickened endometrium (Figure 5C and Figure S6). Statistical analysis demonstrated that the number of glands in the HCur-gel group was significantly higher than that in the IUA group (*P *<* *0.05), similar to the sham surgery group. The recovery of glands in the Gel group and NCHA group was comparatively poorer, with less disparity compared to the IUA group (Figure 5D). Additionally, the endometrial thickness in the LCur-gel group and HCur-gel group was significantly higher than that in the IUA group (*P *<* *0.01), and NCHA exhibited a certain degree of restorative effect on endometrial thickness (Figure 5E). CD31 serves as a crucial marker for vascular endothelial cells [40, 41], and the statistical findings revealed that the positive area in the Gel group, LCur-gel group and HCur-gel group were significantly higher than that in the IUA group (*P *<* *0.01), while there was no notable difference between the NCHA group and the IUA group (Figure 5F and H). In summary, Cur-gel had a superior effect on endometrial regeneration and blood supply compared to NCHA. HCur-gel was comparable to the sham-operated group in terms of endometrial recovery.

**Figure 6.**
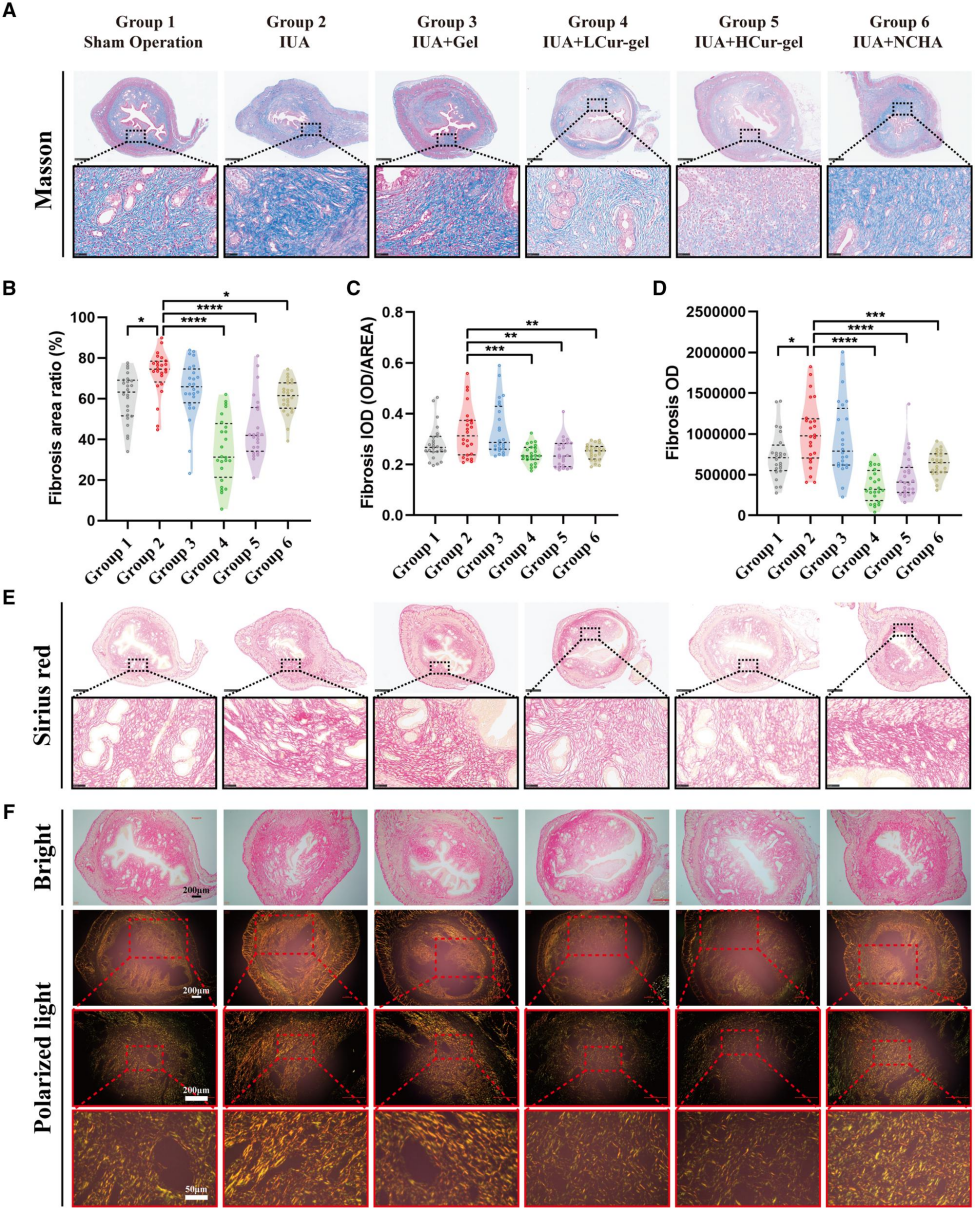
Anti-fibrotic effect of Cur-gels in rats with IUA. (**A**) Corresponding Masson staining. Scale bar: 500 μm, 50 μm. (**B**–**D**) Quantitative analysis of fibrosis. (**E**) Corresponding Sirius red staining. Scale bar: 500 μm, 50 μm. (**F**) Corresponding histological sections under polarized light. Indicating a significant decrease in type I collagen levels in the endometrium after Cur-gels treatment. Scale bar: 200 μm, 50 μm.

The effective reduction of fibrosis generation is a crucial factor in preventing IUAs [42–44]. The average positive expression of TGF-β1 was significantly lower in the LCur-gel group, HCur-gel group and NCHA group compared to the IUA group (Figure 5G and I). The observation of Masson’s stained collagen fibers (blue) clearly indicates that Cur-gel reduces collagen fiber production, and NCHA demonstrates significant anti-fibrotic effects (Figure 6A and Figure S7).

Applying three quantification methods on collagen fibers, the content of collagen fibers in the LCur-gel group and HCur-gel group shows a significant reduction compared to the IUA group (*P *<* *0.01, Figure 6B–D). The Sirius red stained collagen fibers exhibited a similar trend as observed in Masson’s staining (Figure 6E). The sham surgery group and the curettage group did not exhibit the desired significant differences, and in fact, certain quantitative data displayed insignificance. Moreover, the Cur-gel fibrosis data were found to be lower than that of the sham surgery group, and some data even demonstrated a significant decrease. This may be a result of endometrial injury caused by curettage, which leads to a looser arrangement of recently formed endometrial stromal cells and an overall decline in extracellular matrix collagen fiber content. The results align with the anticipated outcomes. An intriguing observation emerged from this study indicating that the NCHA group exhibited superior regeneration and anti-fibrotic effects on the endometrium compared to the Gel group. Theoretically, the expression of CD31 in the NCHA group was expected to surpass that of the Gel group, yet experimental results revealed the contrary. Through a comprehensive review of the literature, it was found that high-molecular weight hyaluronic acid might hinder vascular formation [45, 46], whereas F68 demonstrated a protective effect on damaged microvascular endothelial cells [47], which could elucidate this phenomenon.

The extracellular matrix primarily consists of types I and III collagen. Excessive fibrotic deposition can cause a severe imbalance in the ratio of these collagen types. Type I collagen is characterized by a thick and tightly arranged bundle structure and is predominantly found in the endometrial connective tissue and scar tissue [48–53]. Picrosirius red staining, under polarized light, enables the differentiation of different collagen fibers (type I collagen appears orange-yellow, while type III collagen appears green). Through polarized light observation, Cur-gel has demonstrated a remarkable reduction in type I collagen, indicating its efficacy in combating stubborn connective tissue. Although NCHA possesses some anti-fibrotic effects, its capability in preventing scar formation is unsatisfactory (Figure 6F).

## Conclusion

*In situ* treatment is ideal for managing IUAs. Our research investigated curcumin’s role in fibrotic diseases and its potential use in treating IUAs. Results showed that curcumin promotes angiogenesis, and reduces fibrotic factors *in vitro*, aiding in endometrial repair and preventing adhesion formation. We utilized F127/F68 thermosensitive hydrogel to enhance curcumin’s solubility and meet the requirements of preventing adhesion. The hydrogel has low viscosity at room temperature, facilitating clinical use, and transforms into a semi-solid state at body temperature, remaining in the uterine cavity. *In vivo* experiments demonstrated the effectiveness of the Cur-gel. The results revealed that using a physical barrier alone partially prevents IUAs but has limited efficacy in counteracting connective tissue hyperplasia and restoring endometrial functionality. However, the Cur-gel effectively restores endometrial function and significantly reduces the deposition of type I collagen fibers, thereby reducing abnormal connective tissue proliferation.

In conclusion, we developed a safe and efficient Cur-gel with remarkable anti-IUA effects, surpassing commonly used anti-adhesive agents. This research has the potential to address the postoperative prevention of IUAs and offers new perspectives for the development and application of natural drugs in anti-adhesion therapies for various conditions.

## Supplementary Material

rbae043_Supplementary_Data
