# Supplementary material for: Amorphous curcumin-based hydrogels to reduce the incidence of post-surgical intrauterine adhesions
Source: Regen Biomater. 2024 Apr 24;11:rbae043. doi: 10.1093/rb/rbae043 (PMC11110854; doi:10.1093/rb/rbae043)
Supplement: rbae043_Supplementary_Data [file rbae043_supplementary_data.docx]

# Supporting Information

Amorphous Curcumin-based Hydrogels to Reduce the Incidence of Post-Surgical Intrauterine Adhesions

Wenya Zhang ^a^, Yuxin He ^a^, Yun Chu ^b^, Yuanxin Zhai ^b^, Song Qian ^b^, Xinhui Wang ^a,b^, Pengju Jiang ^a^, Pengfei Cui ^a,^*, Yin Zhang ^c,^*, Jianhao Wang ^a,^ *

a School of Pharmacy, Changzhou University, Changzhou 213164, P. R. China

b Jiangsu Trautec Medical Technology Co., Ltd, Changzhou 213200, P. R. China

c Department of Gynecology, Changzhou Traditional Chinese Medicine Hospital, Changzhou 213004, P. R. China

*Correspondence: cuizy1990@cczu.edu.cn for Dr. Cui Pengfei; 13815075985@163.com for Prof. Zhang Yin; minuswan@cczu.edu.cn for Prof. Wang Jianhao.

Table S1. The gelation temperature of different hydrogel formulations.

| F127/F68 (wt%) | Gel Temperature (℃) | F127/F68 (wt%) | Gel Temperature (℃) |
| --- | --- | --- | --- |
| 20/4 | 24 | 22/5 | 22 |
| 20/5 | 26 | 23/3 | 19 |
| 20/5.5 | 29 | 23/4 | 19 |
| 20/6 | 32 | 23/5 | 19 |
| 21/4 | 24 | 24/3 | 19 |
| 21/5 | 24 | 24/4 | 17 |
| 21/5.5 | 26 | 24/5 | 19 |
| 21/6 | 26 | 24/6 | 19 |
| 22/4 | 20 | 25/4 | ≤15 |


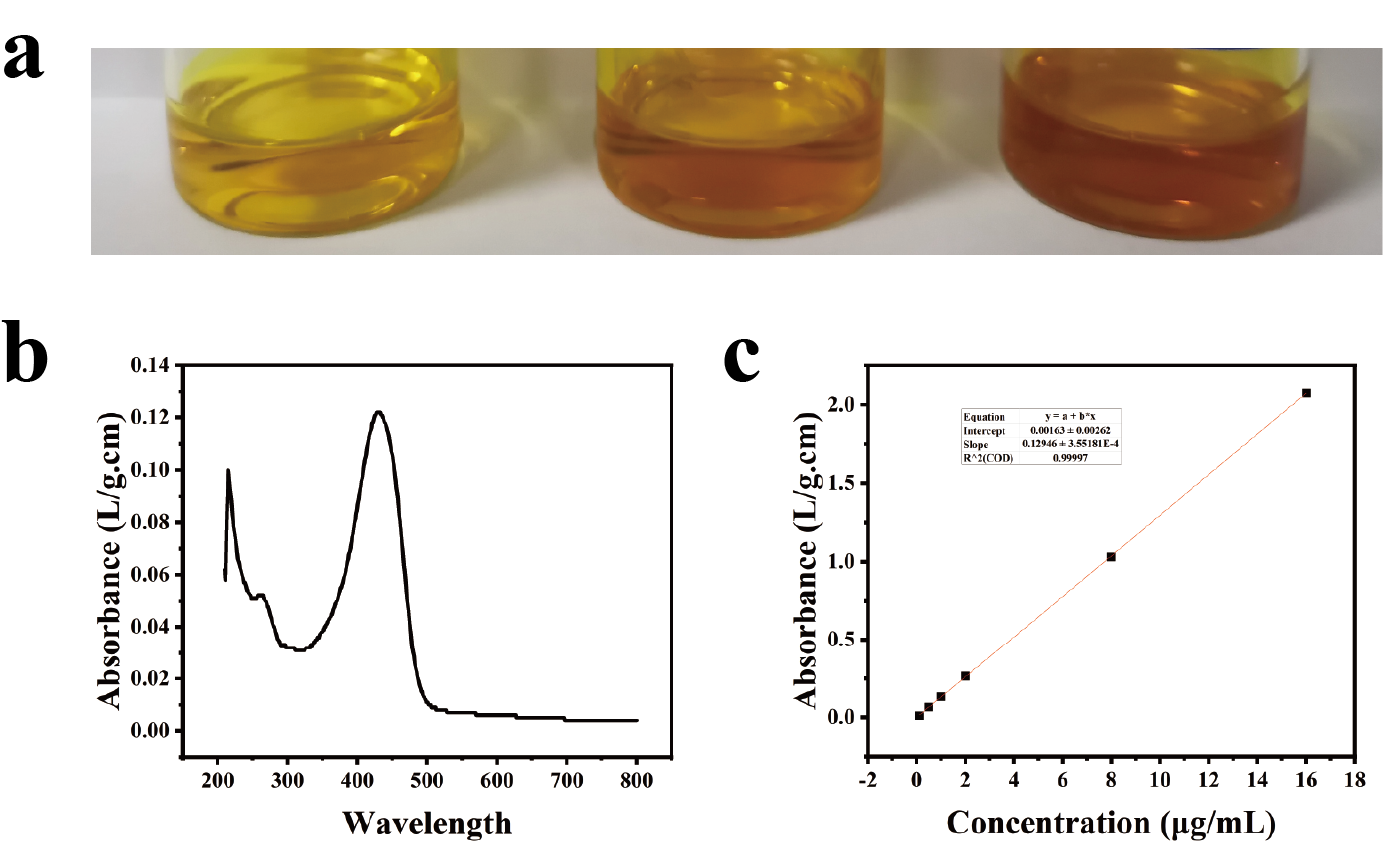


Figure S1. a) Images of Cur-gel dissolved in different concentrations of curcumin. From left to right, 5mg, 10mg, and 40mg of curcumin were added to 5mL of gel and stirred for 24 hours, then centrifuged to remove undissolved curcumin. b) Ultraviolet-visible absorption spectrum of curcumin in anhydrous ethanol. c) UV-Vis calibration curve of known amounts of curcumin with the corresponding linear fit (red curve, λ max = 426 nm). Under these conditions, the maximum dissolution concentration of curcumin was 1.369 mg/mL.


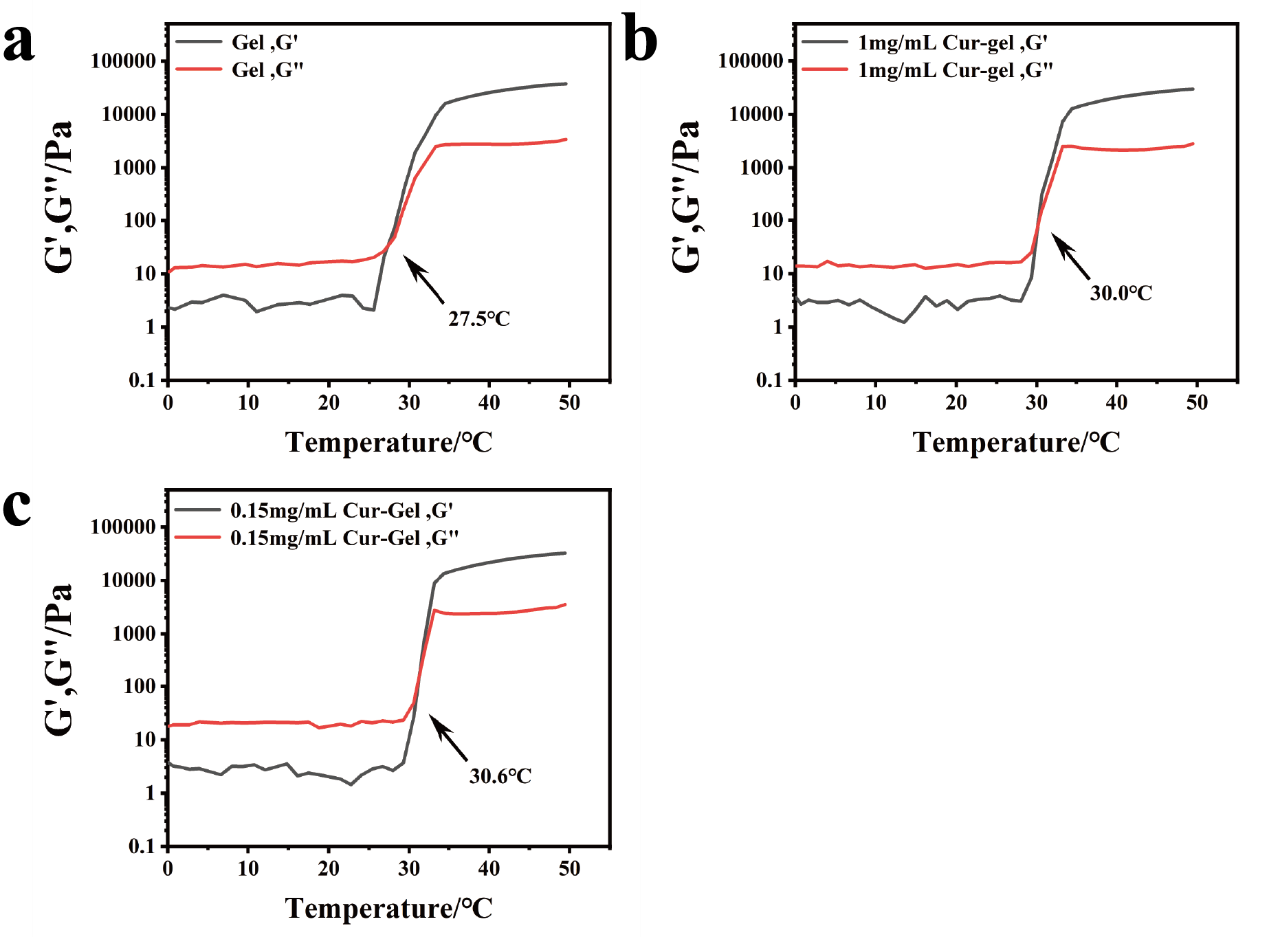


Figure S2. a-c)The effect of temperature on G′ and G″of Gels.


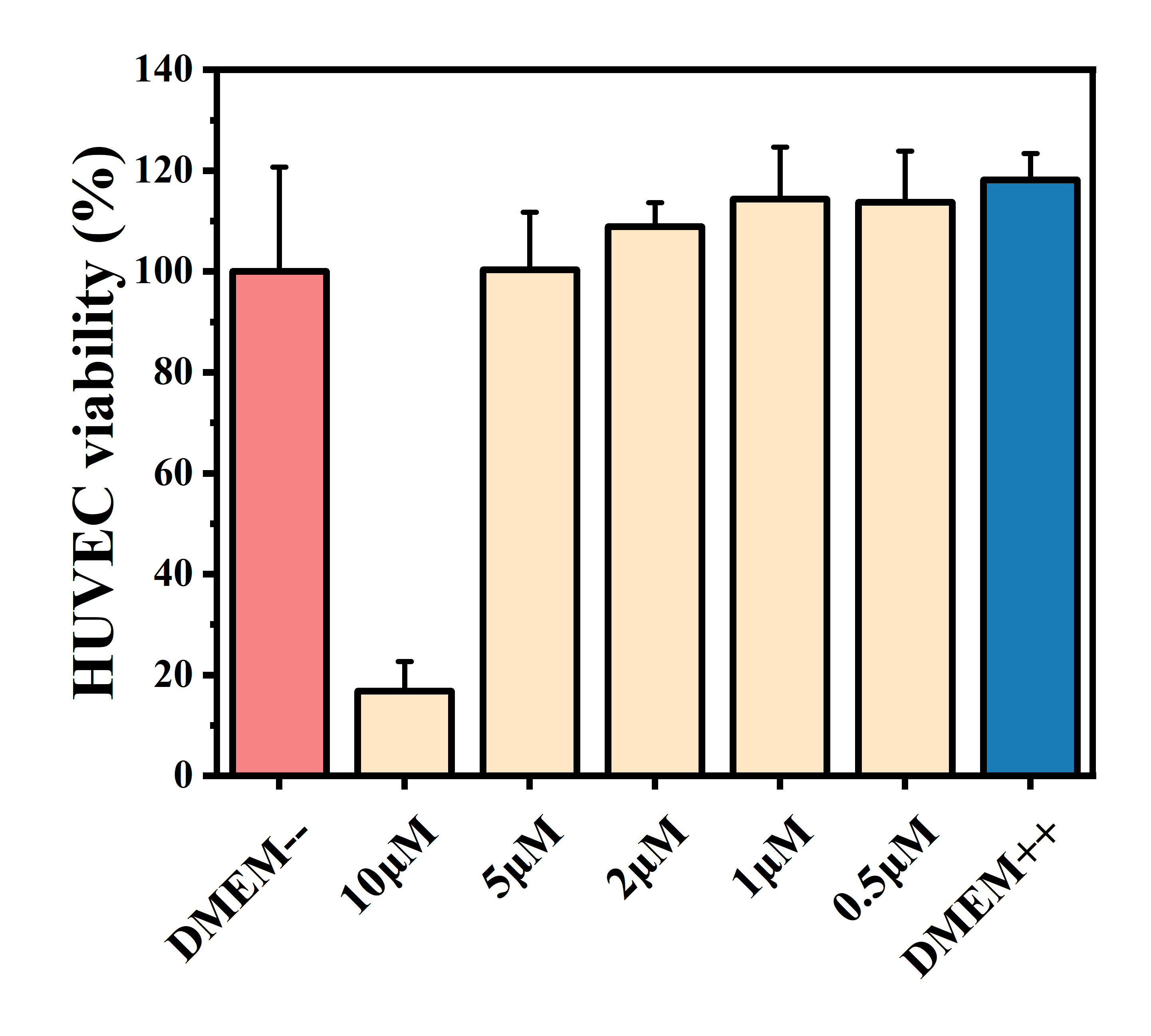


Figure S3. The viability of HUVEC is affected by varying concentrations of curcumin.


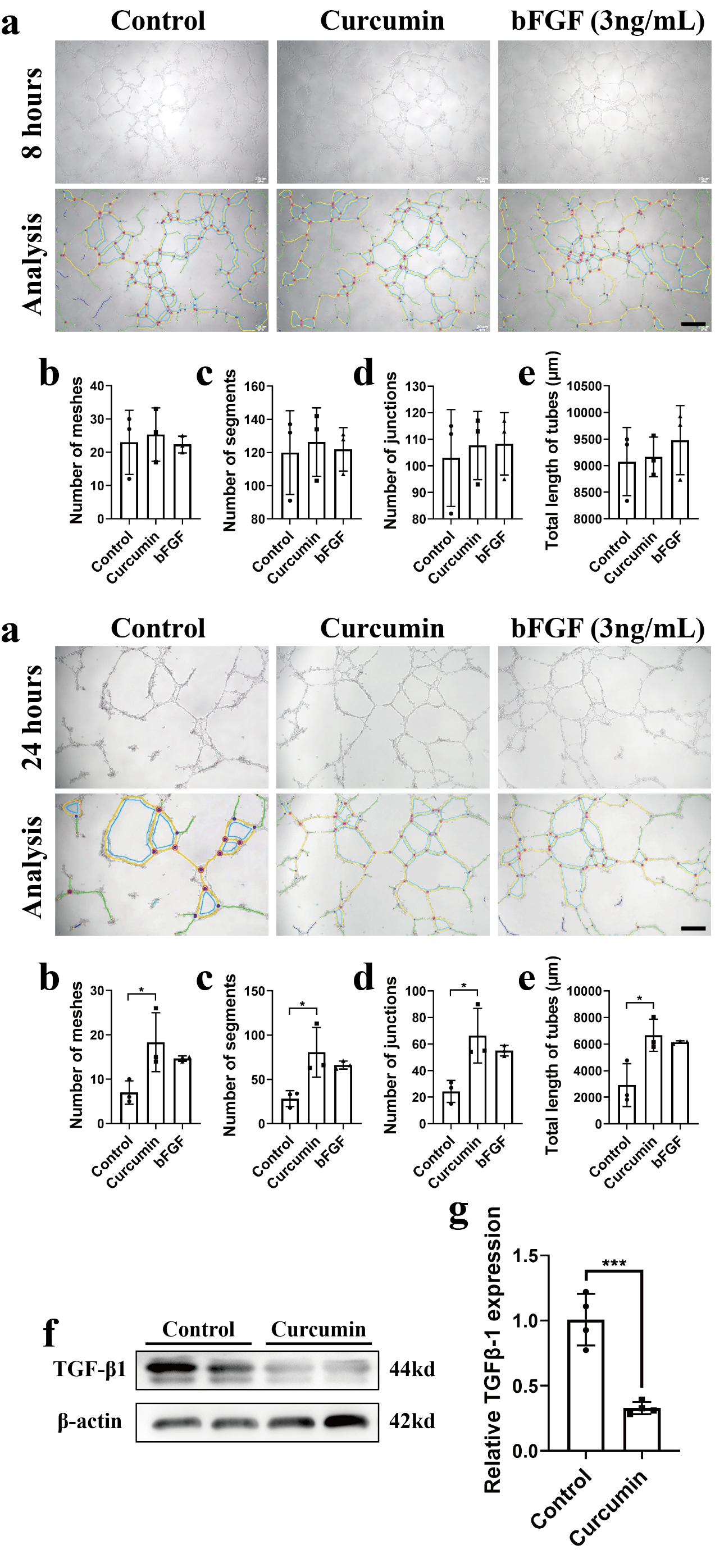


Figure S4. a) Measurement of angiogenesis with the tube formation assay. 1×104 HUVEC cells were suspended in a medium and loaded onto Matrigel. After incubation at 37℃ for 8h, tube formation was directly analyzed in each well using an inverted microscope. The photomicrographs were analyzed using Angiogenesis Analyzer (ImageJ). b-e) Quantitative analysis of capillary tube formation parameters using the Angiogenesis Analyzer for ImageJ. The parameters include the number of meshes, the number of segments, the number of junctions, and the total length of the tube.


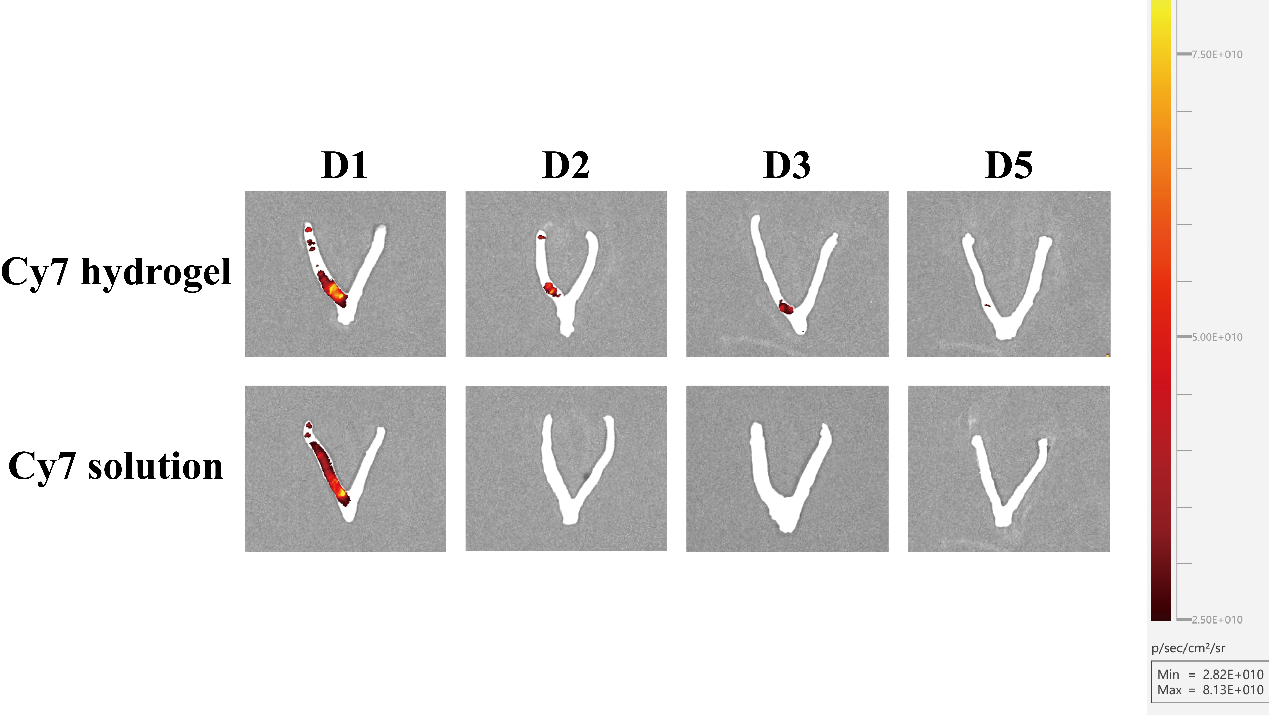


Figure S5. Representative fluorescence images of rat uterus after injection of gel and solution. The right uterus was injected with gel (or solution), while the left one was not treated.


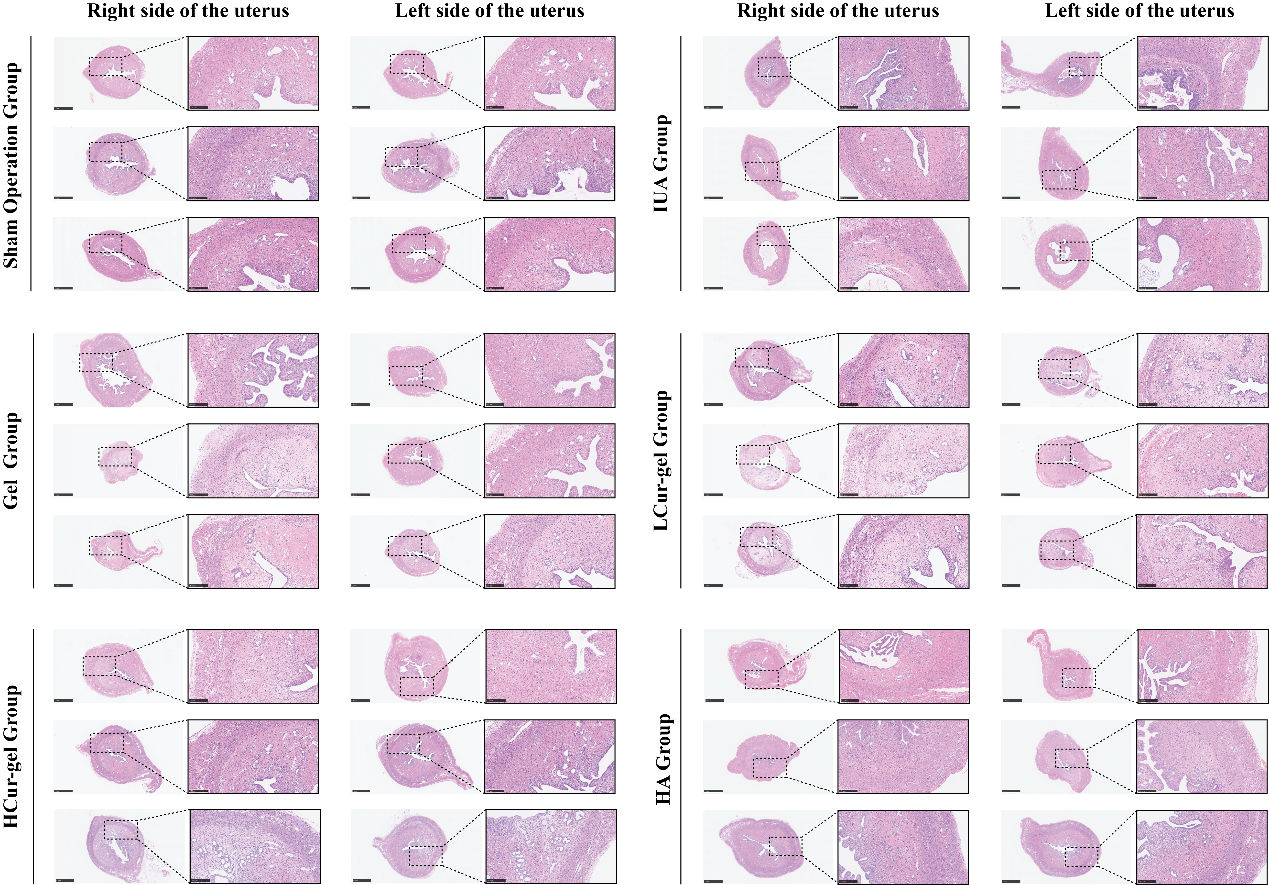


Figure S6. Corresponding HE staining. Scale bar: 1mm, 250μm.


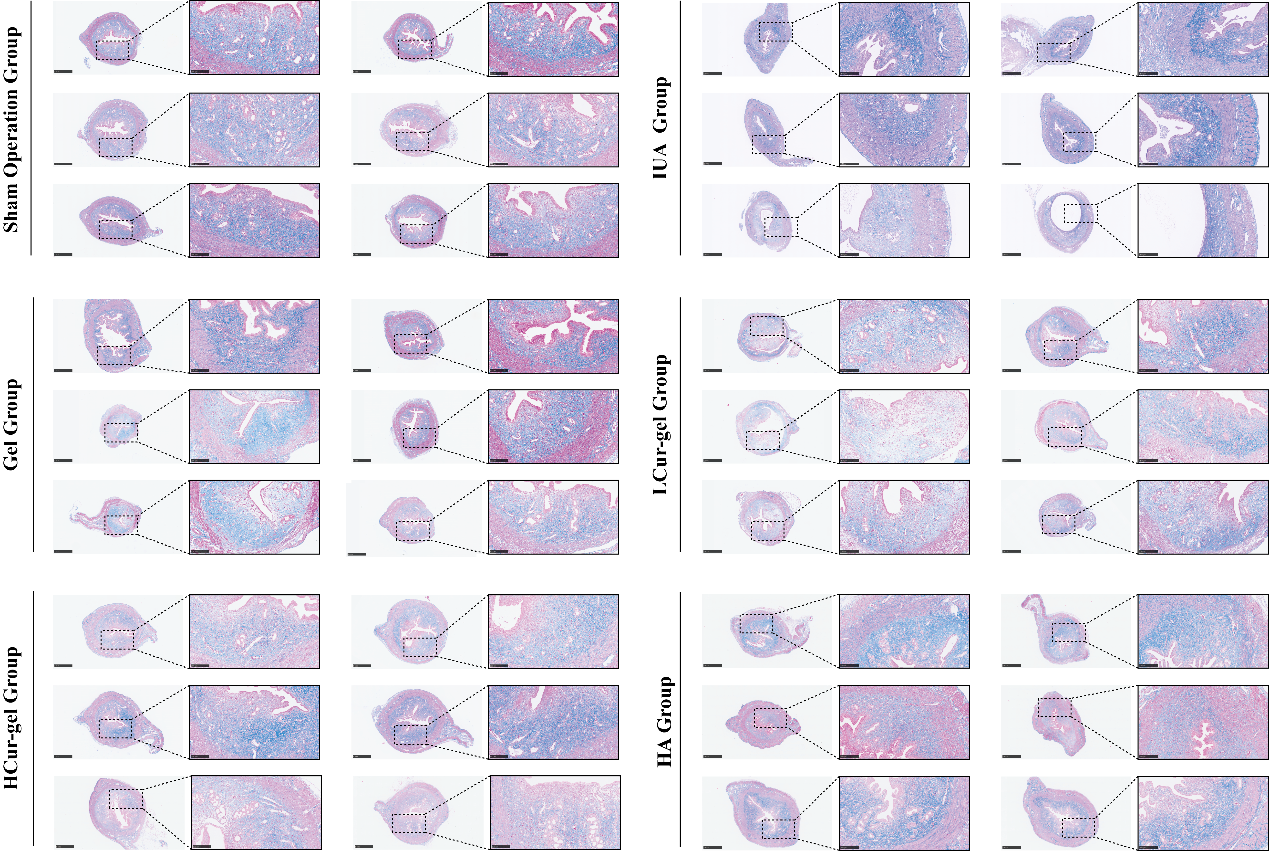


Figure S7. Corresponding Masson staining. Scale bar: 1mm, 50μm.


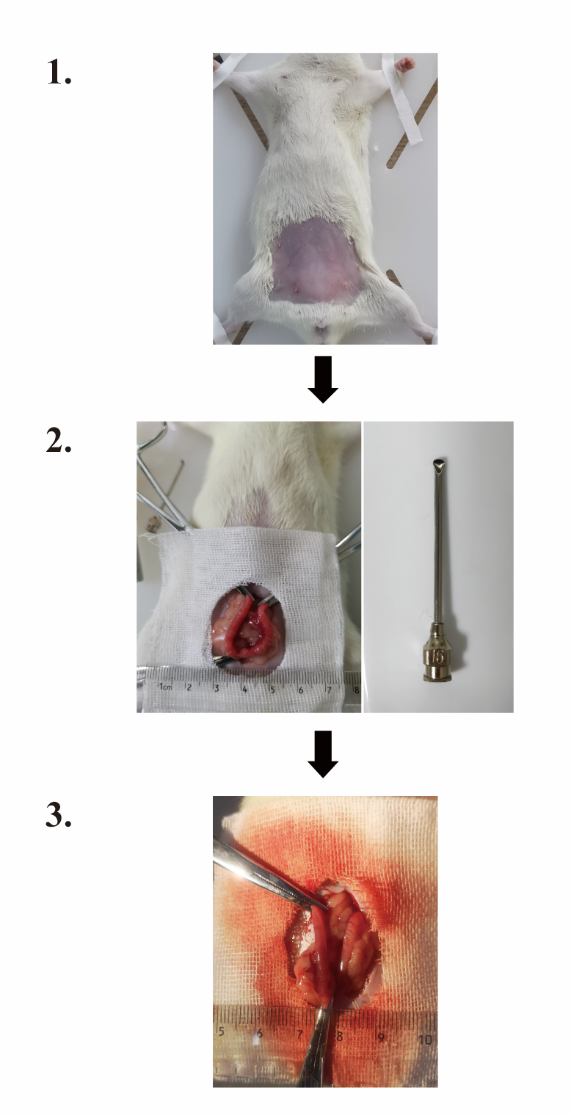


Figure S8. Diagram of IUA rat model establishment. 1)Anesthesia and skin preparation. 2) Pick out the two sides of the uterus, using self-made curettage spoon curettage. 3) The right uterus became red and swollen after curettage injury. The left uterus was not curettage.


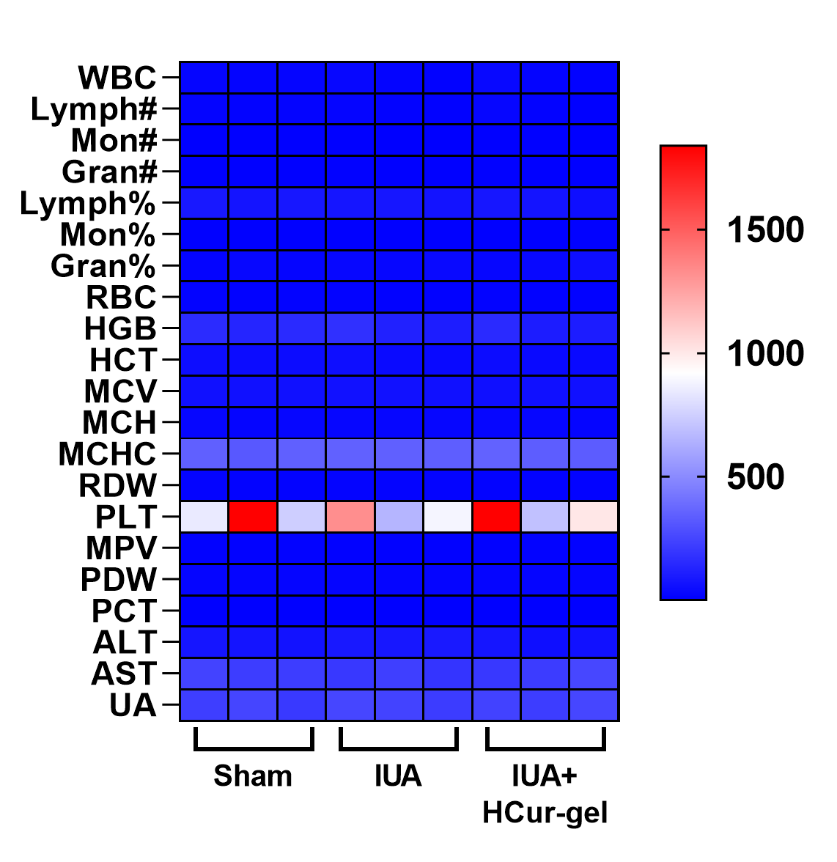


Figure S9. Heat maps displaying blood routine, liver injury (AST and ALT), and kidney injury (UA) indicators for different groups.
